# Recurrent secondary postpartum hemorrhages due to placental site vessel subinvolution and local uterine tissue coagulopathy

**DOI:** 10.1186/1471-2393-14-80

**Published:** 2014-02-21

**Authors:** Pavol Zubor, Karol Kajo, Karol Dokus, Stefan Krivus, Lubomir Straka, Kristina Biskupska Bodova, Jan Danko

**Affiliations:** 1 Department of Obstetrics and Gynecology, University Hospital Martin, Kollarova 2, Martin 036 01, Slovakia; 2 Department of Pathology, University Hospital Martin, Kollarova 2, Martin 036 01, Slovakia; 3 Department of Obstetrics and Gynecology, Jessenius Faculty of Medicine, Comenius University, Kollarova 2, Martin 036 01, Slovak Republic

**Keywords:** Puerperium, Secondary postpartum hemorrhage, Vessel subinvolution, Coagulopathy

## Abstract

**Background:**

Postpartum hemorrhage (PPH) represents a serious problem for women and obstetricians. Because of its association with hemorrhagic shock and predisposition to disseminated coagulopathy, it is a leading cause of maternal deaths worldwide. Furthermore, the jeopardy of PPH is rising with the secondary form of PPH occurring between 24 hours and 6 weeks postpartum, when women are already discharged home. The causes of this pathology are severe inflammation (endometritis), inherited coagulation disorders, consumptive coagulopathy, and retained products of conceptions. Others are of rare occurrence, such as vessel subinvolution (VSI) of the placental implantation site, uterine artery pseudoaneurysm, or trauma.

**Case presentation:**

We present a rare form of recurrent secondary postpartum hemorrhage in a woman after uncomplicated cesarean delivery, with review of the literature linked to the management of this situation originating in the rare local VSI in the placental implantation site, defective decidual homeostasis, and coagulopathy confined to the uterus.

**Conclusion:**

The placental site VSI is one of the rare causes of secondary PPH, and this situation is frequently underdiagnosed by clinicians. The histological confirmation of dilated “clustered”-shaped myometrial arteries partially occluded by thrombi of variable “age” together with the presence of endovascular extravillous trophoblasts confirms the diagnosis.

## Background

Postpartum hemorrhage (PPH) represents serious problem for women and obstetricians. Because of its association with hemorrhagic shock and predisposition to disseminated coagulopathy, it is a leading cause of maternal deaths worldwide [1]. The severity of PPH is potentiated by the fact that it is not specifically associated with the mode of delivery (vaginal vs. cesarean section). Furthermore, the jeopardy of PPH is rising with the secondary form of PPH (abnormal excessive bleeding from the birth canal, mostly uterus, occurring between 24 hours and 6 weeks postpartum), when women are already discharged home.

PPH is a widely underestimated obstetric problem with variable occurrence and severity when diagnosed. This is caused by the lack of definitions and describing criteria used for this condition (e.g., quantification of blood loss, variable cutoff limits for estimated blood loss, and linkage to the mode of delivery). In general, the incidence of PPH is approximately 5%-20% of labors [1,2], with the highest rates in developing countries. The WHO evidence is showing the wide geographic variability in PPH; for example, in Africa, it accounts for 33.9% of maternal deaths (interval 13.3-43.6%), and in Asia, 30.8% (intercountry interval: 5.9-48.5%). Apart from this major problem in the “third world” countries, the rising worries are observed recently also in developed countries like Australia, Belgium, Canada, France, Great Britain, or USA, which are showing increased incidence of PPH (from 1.9% to 2.8%) [3,4]. The causes of this trend are unknown, and we can only hypothesize the preconditions. However, causes can be the recent increase in the overall number of cesarean section, pertinently repeat cesarean section or IVF techniques with an increase in morbidly attached placenta pathologies. In general, it is the change in health-care management about pregnant women and labor conditions/circumstances. However, the common etiologies remain, such as placental abruption, transverse or classical cesarean delivery, manual placental extraction, uterine hypotony/atony, severe inflammation (endometritis), inherited coagulation disorders, consumptive coagulopathy, and retained products of conceptions [4]. Others are of sporadic occurrence and play an important role such as vessel subinvolution (VSI) of the placental implantation site [5,6], trauma, or uterine artery pseudoaneurysm, where the affected vessel wall does not allow adequate contraction and involution. Under the physiological situation, there is spontaneous thrombi and fibrotic closure of the utero-placental vessels in the normal postpartum period. Involution process brings the vessels back to the nongestational state. Failure in this process may be associated with the transient reorganization of the vessel thrombi and their partial occlusion that can lead to severe profuse or intermittent vaginal bleeding after delivery [7], with rapid cardiovascular collapse necessitating sometimes urgent hysterectomy [8,9]. As mentioned previously, the wicked situation occurs when PPH is presented in its secondary form, even if it affects only 1-2% of postnatal women. This low incidence of secondary PPH and linkage to maternal morbidity rather than mortality was the reason for the little attention among obstetricians, but it is recently gaining importance and interest with the understanding of VSI pathology and maternal deaths because of this condition [10]. The primary danger for patient is that bleeding in the majority occurs between 1 and 2 weeks after delivery [11,12] when patient is often home and unaware that the hemorrhage is significant and potentially life threatening.

In this paper, we present a rare form of recurrent secondary postpartum hemorrhage in a woman after uncomplicated cesarean delivery, with review of the literature linked to the management of this situation originating in the rare local VSI in placental implantation site, defective decidual homeostasis, and coagulopathy confined to the uterus.

## Case report

**Figure 1:**
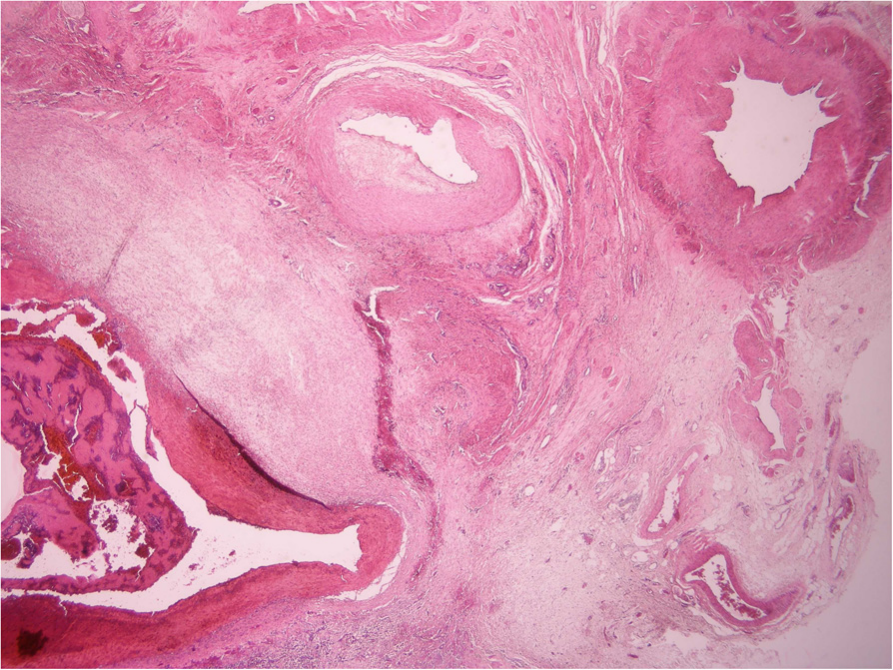
**Local placental site vessel subinvolution (right above) with active bleeding (left down).** (H&E staining, magnification: ×100)

**Figure 2:**
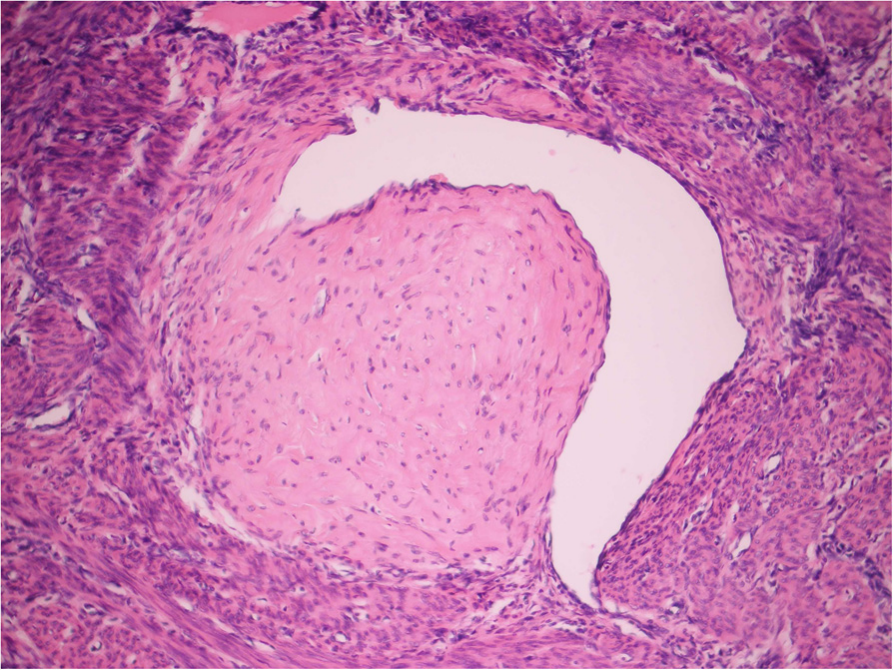
Detailed view on subinvoluted vessel with partial obliteration by thrombi (H&E staining, magnification: ×400).

**Figure 3:**
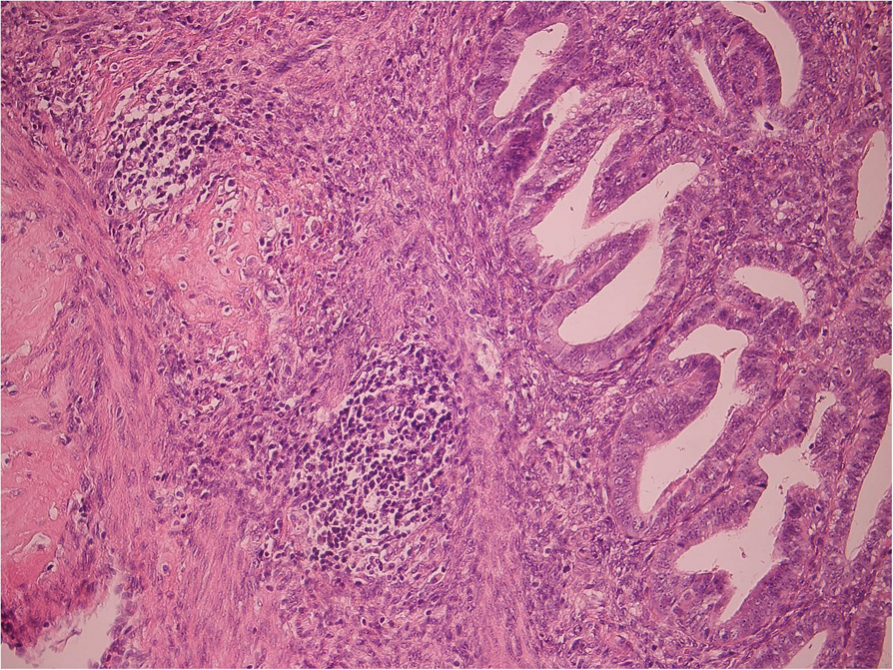
Lymphoid (lympho-plasmocytic) inflammatory infiltration on myometrial and basal endometrium border (H&E staining, magnification: ×200).

A 24-year-old nulliparous primigravida woman was referred to our department in 40 + 3 weeks’ gestation for delivery with history of regular uterine contractions and fetal breech presentation (cervix dilated to 6 cm, presenting part above the interspinal line). She underwent uncomplicated cesarean section (operative technique sec. Pfannenstiel-Geppert, uterus closed by double-layer interlocking separate resorbable sutures, followed by continuous suture of the visceral peritoneum) because of arrest of descent and signs of fetal intrauterine hypoxia. The estimated blood loss was 500 ml, and a prophylactic dose of the first-generation cephalosporin was administered intravenously. Antenatal course was uneventful, except untreated mild pregnancy anemia (hemoglobin (Hb): 102 g/L). Patient was discharged home on the 5th day postpartum with Hb level of 91 g/L, normal coagulation parameters, and a recommendation for iron supplementation and follow-up of blood count. On the 27th day postpartum, she experienced profuse vaginal bleeding with drop in the hemoglobin level (70 g/L), but physical and transvaginal uterine ultrasound examinations failed to reveal the cause (regular uterine involution, no signs of the retained placental tissue, or a visible anomaly of the cesarean scar). Conservative management with blood transfusion units (total volume 670 ml) and uterotonics (oxytocin, methylergometrine) was helpful to stabilize the patient, and she was discharged home within 4 days. Vaginal and cervical microbiological culture has showed disturbed vaginal flora with bacterial colonization of species: *Escherichia coli,* Corynebacterium species*,* Candida species, and *Streptococcus agalactiae* group B. However, recommended antibiotics therapy was not administered because of patient carelessness. On the 43rd day after delivery, she was readmitted with recurrent severe vaginal bleeding (Hb 86 g/L). After initial blood transfusions (680 ml in total), the patient underwent hysteroscopy with curettage, showing no retained products of placental tissue or cesarean scar dehiscence. She was discharged home with blood count of 118 g/L, normal systemic coagulation parameters, and the recommendation of combined estrogen-progestin therapy (estrophem/2 mg/+ duphaston/dydrogesterone/10 mg daily) for early endometrium recovery. Despite this, on the 63rd day following delivery, the patient came back to the hospital with heavy life-threatening vaginal bleeding, signs of hemorrhagic shock (Hb level 61 g/L), and symptoms of hemodynamic instability (sweaty, pale looking, hypotension 90/50 mmHg, and tachycardia 110 b.p.m.). She underwent emergency abdominal supravaginal hysterectomy. Macroscopic examination of the lower uterine segment showed no signs of hysterorrhaphy dehiscence during explorative laparotomy. Similarly, microscopic examination of the uterus and placental implantation site did not confirm the scare dehiscence but revealed large, dilated arteries containing partially occluding thrombi; trophoblastic cells within and surrounding the spiral arteries confirmed by immunohistochemistry; endometrial simplex hyperplasia; and infection (diffuse lymphomonocyte infiltrate) of the implantation site demonstrated by the presence of *Escherichia coli*, *Corynebacterium species*, *Candida species*, and *Streptococcus agalactiae group B* (Figures 1, 2, 3). Additionally, microscopic sections of the blood clot attached to the placental implantation site contained inflamed necrotizing decidua. No retained placenta or placenta accrete was noted on multiple sections. Moreover, the local intravascular coagulation disturbance confined to the uterus was suggested. The postoperative recovery was uneventful, and the patient was discharged after 11 days. One and four months later, she came for follow-up and was healthy.

## Discussion

Postpartum hemorrhage is a serious obstetrics emergency occurring independently from the mode of delivery. The traditional definition of PPH is based on quantification of blood loss but has variable criteria and several limitations across countries or obstetrics national societies. In general, for mild PPH, a blood loss >500 ml and a severe blood loss >1000 ml are considered after spontaneous delivery [13] or above 1000 ml in cesarean section [3]. Regardless of definitions used, only a visual assessment of blood loss is inaccurate [14], and many postpartum women are subsequently underdiagnosed/overdiagnosed for PPH. Thus, alternative blood loss assessment is a check for a drop in hematocrit of 10% counted before and after delivery [15]. The bleeding after labor can occur within 24 hours (primary form of PPH) or, later, from 24 hours after delivery until 6 weeks postpartum (secondary form) of PPH.

During the process of physiological subinvolution, bleeding from the uterus is regulated by several factors and mechanisms, for example, myometrial contraction and local decidual and systemic coagulation factors leading to the minimalization of blood loss after delivery. Moreover, at the end of the third trimester, the extravillous endovascular trophoblast (EVCT) for maternal endothelial cells in myometrial vessels located in the placental site is starting to be replaced. With this vascular involution, change is playing a role also in the occlusive form of the intima layer thickening via its fibrotic transformation, local endarteritis, thrombotization, and regeneration of the internal elastic lamina of the uterus vascular system.

In pathology, for example, with abnormal complement component activity or with the presence of long-lasting expression of apoptotic genes as Bcl-2 inhibiting apoptosis and supporting cell survival, [16] VSI develops (luminal partial occlusion of the uterine vessels) in the previous placental site implantation. This would result in temporary occlusion of the vessels (mainly spiral arteries in the superficial myometrium at the placental implantation site) where the newly formed thrombi undergo resorption within a period of 2–3 weeks, which can lead to recurrent uterine bleeding and enlarged and boggy uterus. The possibility of such phenomenon is becoming more obvious when bleeding reoccurs in regular periods. In our case, the uterine bleeding recurred in a period of 15–20 days (27th, 43rd, and 63rd day postpartum). The severity of bleeding can be augmented by the presence of active inflammation (confirmed also in our case), which may enhance through the production of proinflammatory factors of the local tissue coagulopathy [17] and switch to the chronicity with the development of chronic disseminated intravascular coagulation [18,19]. Such “terrain” is predisposed to secondary PPH. The situation observed also by our study.

To diagnose the placental site VSI with the presence of local site inflammation and tissue coagulopathy is difficult. The exact directed histological examination of bioptic material from the previous placental site can be helpful. The presence of vascular subinvolution can be confirmed by the finding of EVCT. Unfortunately, we did not observe this in our histological specimens that were obtained from hysteroscopy and curettage. In this situation, we have many other clinicians working under pressure and focused more on finding potential myometrium scar dehiscence and observing the condition of the endometrium (hyperplasia after hormonal replacement therapy). Omitting detection of EVCT and other possible helpful markers as immunostaining positivity for cytokeratin profile, inhibin alpha, Mel-CAM, Bcl-2, elastin, complement components, or human placental lactogen is a mistake as they are enriching the information for the direct diagnosis of VSI. Moreover, the histological evidence of VSI is also the presence of complement cascade markers (C1q, C3d, C4, and C9) or immunoglobulins G, A, and M [20]. However, although all of these markers can be evaluated, they did not explain the basic molecular mechanism of placental site subinvolution. Surgical pathologist must therefore be aware of the major histopathologic findings of VSI in every postpartum curettage or hysterectomy specimens.

The exact pathophysiology of VSI is not known. Some suspect an immune component leading to abnormal interaction between the maternal uterine cells and fetal trophoblast [5]; others, as mentioned above, altered complement components activity or the presence of long-lasting expression of apoptotic genes (e.g., Bcl-2), inhibiting apoptosis [16]. However, after the review of the literature, there is one specific situation that is related to secondary postpartum hemorrhage and published in MEDLINE. The search in this register (June 2011) under keywords “secondary or delay or unusual cause of postpartum hemorrhage” showed 18 papers dealing with secondary postpartum hemorrhage where 9 papers [7,10,21-27] reported cases of secondary PPH after cesarean section with two major conclusions: placental site VSI or uterine artery pseudoaneurysm. To explain how cesarean section enhances the higher occurrence of VSI is difficult. One may only speculate that some mechanic (e.g., altered myometrium contraction and involution, fibrotic-proliferation in scar), immune (autoimmune and healing processes, reaction to suturing material used, local viral or bacterial inflammation, role of lymphomonocytic and cytokine activity), or molecular mechanisms like overexpression and underexpression of growth factor genes involved in the uterine scarring process, genes modifying the decidua replacement by regular endometrium, collagen deposition in the vessels of wound site, platelet aggregation, factor XIII activity, and gene expressions of pro-angiogenic and anti-angiogenic factors and receptors at the maternal-fetal interface are playing a role with a consequent secondary postpartum hemorrhage [28-31].

Although the incidence of secondary postpartum hemorrhage after cesarean section is very low (app. 0.1%) [22], therapeutic management is close to primary PPH and requires coordination and multidisciplinary care, aiming the immediate hemodynamic stabilization of the patient, depleted blood volume, and development of coagulopathy. Treatment usually falls into one of two options: surgical evacuation of the uterine cavity or medical treatment [32]. Often, blood and plasma unit transfusion is required. Speculum examination of the cervix and the lower genital tract to exclude possible lacerations is obligatory. Furthermore, uterine ultrasound is mandatory to exclude a possibility of the retained placental tissue [33]. In differential diagnosis, it is necessary to exclude vaginal bleeding because of the severe endometritis, retained placental tissue, or gestational trophoblastic disease, where laboratory findings of inflammatory markers (e.g., CRP, leucocytes), positive blood and vaginal cultures, elevated βHCG levels, and expert ultrasound examination [33,34] are essential for the adequate diagnosis.

## Conclusions

In conclusion, we can say that placental site VSI is one of the rare forms of secondary PPH and is frequently underdiagnosed by clinicians. The histological confirmation of dilated and “clustered”-shaped myometrial arteries partially occluded by thrombi of variable “age,” together with the presence of endovascular extravillous trophoblast, confirms the diagnosis. However, this is done mainly after the urgent hysterectomy. Furthermore, we believe that this type of secondary PPH is of idiopathic than of iatrogenic cause, and our previously published [35] data prove that there are no known predictive factors for this pathology (e.g., clinical assessment with/without transabdominal ultrasound). The obstetricians and caregivers are demanding for an early recognition of severe forms of a secondary PPH, thus emphasizing that VSI in the placental implantation site could be beneficial for targeted therapy and may preserve woman’s fertility.

### Consent

Written informed consent was obtained from the patient for publication of this Case report and any accompanying images. A copy of the written consent is available for review by the Editor of this journal.

## Competing interests

The authors report no declarations of interest.

## Authors’ contributions

PZ and KD contributed equally to write up of this case report. SK and KBB were involved in the clinical management of this patient. KK and LS were involved in histopathological analysis. JD interpreted the data and critically revised the manuscript. All authors have read and approved the final manuscript.

## Pre-publication history

The pre-publication history for this paper can be accessed here:

http://www.biomedcentral.com/1471-2393/14/80/prepub
